# Drug Resistance in Late-Stage Epidermal Growth Factor Receptor (EGFR)-Mutant Non-Small Cell Lung Cancer Patients After First-Line Treatment with Tyrosine Kinase Inhibitors

**DOI:** 10.3390/ijms26052042

**Published:** 2025-02-26

**Authors:** Ching-Yi Lee, Shih-Wei Lee, Yi-Chiung Hsu

**Affiliations:** 1 Department of Internal Medicine, Tao Yuan General Hospital, Taoyuan 33004, Taiwan; chestman9@gmail.com; 2 Department of Biomedical Sciences and Engineering, National Central University, Taoyuan 320317, Taiwan; 3 Department of Nursing, Yuanpei University of Medical Technology, Hsinchu 30015, Taiwan; 4 Center for Astronautical Physics and Engineering, National Central University, Taoyuan 320317, Taiwan; 5 Department of Medical Research, Cathay General Hospital, Taipei 106438, Taiwan

**Keywords:** epidermal growth factor receptor (EGFR), non-small cell lung cancer (NSCLC), tyrosine kinase inhibitors (TKIs), next-generation sequencing (NGS)

## Abstract

The development of tyrosine kinase inhibitors (TKIs) for late-stage epidermal growth factor receptor (EGFR)-mutant non-small cell lung cancer (NSCLC) represented a drastic change in the treatment of late-stage lung cancer. Drug resistance develops after a certain period of first-line TKI treatment, which has led to decades of changing treatment guidelines for EGFR-mutant NSCLC. This study discussed the potential mechanisms of drug resistance against first-line TKI treatment and potential successive treatment strategies. Next-generation sequencing (NGS) may play a role in the evaluation of drug resistance in first-line TKI treatment. Emerging combination regimens and ongoing trials were discussed. Potential future strategies for treatment and for the management of drug resistance were proposed in this study.

## 1. Introduction and Background and Evolution of EGFR-Targeted Therapy

Lung cancer is classified by histology: 15% is small-cell lung cancer, and 85% is non-small-cell lung cancer. About half of the non-small-cell lung cancer is adenocarcinoma and late-stage lung adenocarcinoma patients were previously treated with conventional chemotherapy and could have overall survival rates of less than one year. The presence of activating epidermal growth factor receptor (EGFR) mutations sensitive to EGFR tyrosine kinase inhibitors (TKIs) was originally noted in lung adenocarcinoma patients. In the IPASS study [1] and other studies [2,3], first-line administration of gefitinib, the tyrosine kinase inhibitor (TKI) for epidermal growth factor (EGFR) mutations, to late-stage epidermal growth factor (EGFR)-mutant non-small cell lung cancer patients resulted in increased progression-free survival (PFS) and overall survival (OS). Kim et al. [4] conducted an open-label phase 3 study (INTEREST) at 149 centers in 24 countries, recruiting 1466 patients with pretreated advanced NSCLC, who were randomly assigned to receive gefitinib or docetaxel between 2004 and 2006. The non-inferiority of gefitinib compared with docetaxel was shown, with a hazard ratio of 1.02. Maruyama et al. [5] conducted a phase 3 study (V-15-32) comparing gefitinib with docetaxel in previously treated Japanese patients with NSCLC. Although non-inferiority in overall survival between gefitinib and docetaxel was not demonstrated according to the predefined criteria, statistically, there was no significant difference between the overall survival rates of the two groups.

Regarding demographic and clinical factors, Asian race, female sex, nonsmoking status, and adenocarcinoma were shown to be the predictive factors of the treatment efficacy of gefitinib. A number of studies [6,7,8,9] showed that the oral administration of the EGFR tyrosine kinase inhibitor was at least as effective as a first-line treatment as chemotherapy. In the IPASS trial [1], gefitinib was also associated with a better PFS and objective response rate compared with carboplatin–paclitaxel. The objective response rate in the overall population was significantly higher with gefitinib (43.0% vs. 32.2%; odds ratio: 1.59; 95% CI: 1.25 to 2.01; *p* < 0.001). In the phase 3, open-labeled IPASS trial, patients receiving first-line treatment with gefitinib had longer progression-free survival (PFS) compared with patients receiving conventional chemotherapy of carboplatin–paclitaxel in the mutation-positive subgroup (hazard ratio for progression: 0.48; 95% CI: 0.36 to 0.64; *p* < 0.001). The objective response rate (ORR) was 71.2% with gefitinib compared with 47.3% with carboplatin–paclitaxel in the mutation-positive subgroup (*p* < 0.001). In Japan, Maemondo et al. [10] recruited 230 eligible untreated metastatic EGFR-mutant NSCLC patients and randomly assigned them to the gefitinib group and the chemotherapy group. The gefitinib group had significantly longer PFS compared with the chemotherapy group (10.8 vs. 5.4 months; *p* < 0.001). However, in the NEJ002 study [11], mean survival time was not statistically different between the gefitinib group and the chemotherapy group, which may be due to the high cross-over use of gefitinib in the chemotherapy group. In another open-label, phase 3 study (WJTOG3405) [12], 172 eligible patients were randomly assigned to the gefitinib group and chemotherapy group. The gefitinib group had longer PFS compared with the chemotherapy group (9.2 months vs. 6.3 months; *p* < 0.0001). Successive discoveries of several generations of EGFR tyrosine kinase inhibitors (TKIs) have opened new horizons for the treatment of late-stage EGFR-mutant NSCLC patients [13,14,15,16,17,18]. Before the age of the third generation TKI, the FDA granted erlotinib for the treatment of NSCLC patients who failed at least one chemotherapy [19].

In the open-label, randomized phase 3 EURTAC trial [13] conducted in Europe, 174 patients with EGFR mutations were enrolled between 2007 and 2011 and randomized into two groups. One group received oral erlotinib (150 mg per day), and the other group received standard chemotherapy. At the data cutoff (26 January 2011), the median PFS was 9.7 months (95% CI: 8.4–12.3) in the erlotinib group, compared with 5.2 months (4.5–5.8) in the standard chemotherapy group. In the OPTIMAL study [14], an open-label, randomized, phase 3 trial conducted at 22 centers in China, 83 eligible patients were enrolled and randomized into two groups. One group received erlotinib, and the other group received standard chemotherapy. The erlotinib group had a significantly longer mean PFS than the chemotherapy group (13.1 [95% CI: 10.58–16.53] vs. 4.6 [4.21–5.42] months; hazard ratio: 0.16; 95% CI: 0.10–0.26; *p* < 0.0001).

Sequist et al. [15] conducted the randomized, open-label phase 3 study LUX-Lung 3. A total of 345 eligible patients with EGFR mutations were enrolled and randomly assigned in a two-to-one fashion. One group received daily oral afatinib (40 mg) and was compared to the other group, which received standard chemotherapy. The median PFS of the patients with common EGFR mutations (*Exon 19* deletions and *L858R* mutations) receiving afatinib was 13.6 months compared with a median PFS of 6.9 months in the chemotherapy group (HR: 0.47; 95% CI: 0.34 to 0.65; *p* = 0.001). Wu et al. [16] conducted the open-label, randomized phase 3 trial LUX-Lung 6 in Asia. A total of 364 eligible patients were randomly assigned (2:1) to receive either daily oral afatinib (40 mg) or standard chemotherapy. The median PFS was 11.0 months in the afatinib group compared with a median PFS of 5.6 months in the chemotherapy group (hazard ratio: 0.28; 95% CI: 0.20–0.39). In July 2013, the FDA granted approval for afatinib, an irreversible inhibitor of the ErbB family of tyrosine kinases, which binds to EGFR, HER2, and HER4, irreversibly inhibiting tyrosine kinase autophosphorylation, for the first-line treatment of EGFR mutation-positive metastatic NSCLC [20]. Wu et al. [18] conducted the randomized, open-label, phase 3 study ARCHER 1050. Between May 2013 and Mar 2015, 452 eligible patients were randomized to receive either dacomitinib or gefitinib. The median PFS in the dacomitinib group was 14.7 months compared with a median PFS of 9.2 months in the gefitinib group (hazard ratio: 0.59; 95% CI: 0.47–0.74; *p* < 0.0001).

The third-generation TKI, osimertinib, was first approved by the FDA [21] for adult patients with metastatic EGFR *T790M* mutation-positive NSCLC. Soria and other FLAURA investigators [22] conducted a double-blind, phase 3 trial in which 556 eligible untreated patients with common EGFR mutations (*Exon 19* deletion or *L858R*) were randomized in a 1:1 ratio to receive either osimertinib or gefitinib. The median PFS in the osimertinib group was 18.9 months compared with a median PFS of 10.2 months in the gefitinib group (hazard ratio for disease progression or death: 0.46; 95% confidence interval [CI]: 0.37 to 0.57; *p* < 0.001). After 2 years of consecutive study and follow-up in accordance with the trial design, the median OS was 38.6 months in the osimertinib group compared with a median OS of 31.8 months in the gefitinib group (hazard ratio for death: 0.80; 95.05% CI: 0.64 to 1.00; *p* = 0.046) [23]. With the significant duration of the response and improved OS compared with the standard TKI regimen that was documented in the FLAURA trial [22,23], the FDA approved osimertinib as a first-line treatment for late-stage EGFR-mutant NSCLC. Despite osimertinib treatment, most patients’ disease progressed. Previous studies, including phase 2 and phase 3 NEJ009 studies [24,25], showed the significantly improved efficacy of gefitinib plus platinum-based chemotherapy compared with gefitinib monotherapy. To investigate the efficacy of osimertinib in combination with chemotherapy, Planchard and other FLAURA2 investigators [26] conducted the phase 3, international, open-label FLAURA2 trial. Between June 2020 and Dec 2021, 557 eligible patients were recruited and randomly assigned to an osimertinib–chemotherapy group and an osimertinib group. An objective response was observed in 83% of the patients in the osimertinib–chemotherapy group and in 76% of those in the osimertinib group; the median response duration was 24.0 months in the osimertinib–chemotherapy group and 15.3 months in the osimertinib group. Due to its significant efficacy benefit, the NCCN has listed osimertinib–chemotherapy combination therapy as a category 1 treatment for late-stage EGFR-mutated NSCLC.

Amivantamab, an EGFR mesenchymal–epithelial transition factor (MET)-bispecific monoclonal antibody binding to the extracellular domains of EGFR and MET, was initially approved by the FDA for adult patients with locally advanced or metastatic NSCLC carrying *Exon 20* insertion (*Ex20ins*) mutations [27]. In the engineered cell lines, patient-derived cells (PDCs), and patient-derived xenografts (PDXs), amivantamab (JNJ-61186372) showed antitumor activity. Yun et al. [28] conducted a study utilizing Ba/F3 cells with overexpression of EGFR *Exon20ins* and compared the antitumor activity of amivantamab (JNJ-61186372) to the first- and third-generation irreversible EGFR TKIs gefitinib and osimertinib. With five distinct *Exon20ins* in the Ba/F3 cells, treatment with amivantamab doses ranging from 0.05 to 1 mg/mL was applied. A significant and dose-dependent decrease in BA/F3 cell viability was observed. In contrast, treatments with gefitinib and osimertinib showed limited antitumor activity. In a phase I, open-label, dose-escalation, and dose-expansion study (CHRYSALIS), Park et al. [29] enrolled eligible post-platinum EGFR *Exon20ins* NSCLC patients and treated them with the recommended dose of 1050 mg of amivantamab (every week for 4 weeks, and then every 2 weeks). In the CHRYSALIS cohort D, the overall response rate was 40%, and the duration of response was 11.1 months. The median PFS was 8.3 months (95% CI: 6.5 to 10.9). Using the large-scale clinico-genomic database LC-SCRUM-Asia [30], 189 patients with non-small cell lung cancer harboring EGFR Exon20 in-frame insertions were identified. Following further classification as near-loop or far-loop insertions of *Exon20*, Okahisa et al. found that osimertinib treatment was associated with a longer PFS in patients with *Exon20ins* near-loop insertions compared with those with far-loop insertions (median: 5.6 vs. 2.0 months; HR [95% CI]: 0.22 [0.07–0.64]). After platinum-based chemotherapy, patients with non-small cell lung cancer harboring EGFR *Exon20* achieved a risk reduction in terms of their PFS and overall survival with treatment of amivantamab in the CHRYSALIS trial compared with LC-SCRUM-Asia patients who were treated with docetaxel, classic TKIs, or immunotherapy.

It is already known that amivantamab administration may cause infusion-related reactions, including chills, dyspnea, flushing, nausea, chest discomfort, and vomiting. To prevent and manage amivantamab-infusion-related reactions, Park et al. [31] developed mitigation strategies and an infusion protocol, including premedication with steroids, antihistamines, and antiemetics. A divided dose of 350 mg of amivantamab was infused on day 1 via the peripheral line, followed by a 700 mg or 1050 mg infusion on day 2. A consecutive loading protocol on days 8, 15, and 22 was implemented. Infusion-related reactions (IRRs) primarily occur early, within the first 60 min of amivantamab infusion. With this mitigation strategy, the practitioners may lessen the severity of IRRs caused by the amivantamab infusion. In the MARIPOSA Trial [32], amivantamab–lazertinib led to a superior PFS and median response duration compared with the osimertinib group in patients with previously untreated or osimertinib-pretreated EGFR-mutated advanced NSCLC. In the eleventh version (2024) and the third version (2025) of the NCCN Clinical Practice Guidelines for NSCLC [33], osimertinib with or without chemotherapy and amivantamab–lazertinib are listed as first-line treatments for adult patients with advanced NSCLC-harboring EGFR Exon 19 deletion or Exon 21 L858R mutations (Table 1).

## 2. Drug Resistance and Mechanisms

With the use of TKIs, patients with EGFR-mutated adenocarcinoma experience the progression of their disease after months or years of treatment. Drug resistance through multiple resistance mechanisms can be categorized into acquired mutations, bypass signaling activation, and phenotypic transformation. The most common acquired mutation is EGFR *T790M*, which increases ATP affinity and reduces the efficacy of first- and second-generation TKIs. In the open-label, exploratory, randomized controlled-phase 2B trial LUX-Lung 7 [34], Park et al. enrolled 319 eligible patients and randomized them into an afatinib group and a gefitinib group. The PFS and time to treatment failure in the afatinib and gefitinib groups were as follows: the PFS was 11.0 months vs. 10.9 months (hazard ratio [HR]: 0.73 [95% CI: 0.57–0.95]); the time to treatment failure was 13.7 months [95% CI: 11.9–15.0] in the afatinib group and 11.5 months in the gefitinib group; and the HR was 0.73 ([95% CI: 0.58–0.92]; *p* = 0.0073). To study the frequency of *T790M* mutations in advanced EGFR-mutant lung adenocarcinoma patients with acquired resistance after first-line EGFR-TKI treatment, Huang et al. [35] enrolled 205 EGFR-mutant stage IIIB-IV lung adenocarcinoma patients. After disease progression, re-biopsies were performed. The *T790M* mutation rate in the re-biopsies was 46.3%. In the subgroup analysis, the study showed that patients harboring *Exon 19* deletions had *T90M* mutation rates of 55.0%, whereas patients harboring *L858R* point mutations had mutation rates of 37.3 (adjusted odds ratio: 2.14; 95% CI: 1.20 to 3.83; *p* = 0.010). In a retrospective observational study in Taiwan [36], 407 patients were enrolled, and re-biopsies of the patients were performed for further *T790M* mutation identification. The overall re-biopsy *T790M* mutation rate was 52.8%. The T790M mutation rates were statistically different based on the previous first-line TIK treatment (gefitinib: 59.9%; erlotinib: 45.5%; afatinib: 52.7%; *p* = 0.037). Further multivariate logistic regression analysis showed that patients with common baseline EGFR mutations, patients treated with gefitinib administration (compared with erlotinib), and patients with longer TKI treatment durations had a higher *T790M* incidence.

A single-country, multicenter, prospective cohort study in Greece, the “LUNGFUL” observational study [37], aimed to evaluate the frequency of acquired *T790M* resistance in late-stage EGFR-mutant NSCLC patients who had received first-generation or second-generation TKIs (erlotinib, gefitinib, and afatinib) and experienced disease progression. The investigators enrolled 96 eligible patients between July 2017 and September 2019, with 94 patients receiving liquid biopsies and the remaining two receiving tissue biopsies. Using the cobas^®^ EGFR Mutation test, *T790M* mutations were detected in 16.0% (15/94) of the plasma biopsies and one tissue biopsy from the primary tumor. Regarding ethnicity, the *T790M* mutation rates are lower in Caucasian patients than those identified in the previously described Asian population study. In a network meta-analysis [38] analyzing *T790M* mutation rates after the first- and second-generation TKIs, 29 studies were included. Most of the *790M* re-biopsy samples were from tissue; four of the studies included tissue and fluid samples, three of the studies included tissue and plasma samples, and three of the studies only collected plasma samples. The *T790M* mutation rate was 33% with afatinib, 49% with gefitinib, and 47% with erlotinib treatment (*p* < 0.001). A slight difference in the acquired *T790M* mutation rates depending on ethnicity was identified, with a rate of 43% in Asian patients and 47% in Caucasian patients. Huang et al. [39] prospectively enrolled 80 eligible late-stage EGFR-mutant lung adenocarcinoma patients. Plasma samples were collected before TKI treatment and every 3 months after the start of treatment. Droplet digital PCR (ddPCR) tests were analyzed to detect *T790M* in the plasma circulating tumor DNA (ctDNA), and the patients were followed until the date of their death or to the end of 2021. Of 80 eligible patients, 75 had progressive disease, and 53/75 (71%) received re-biopsies. A *T790M* mutation was detected in 28/53 (53%) of the samples, while the ddPCR tests detected 23/53 (43%) positive *T790M* mutations. The concordance rate between tissue re-biopsy and plasma ddPCR was 76%.

Combination treatment with first- and second-line EGFR-TKIs and bevacizumab can also lead to the development of *T790M* mutations. In the retrospective multicenter TERRA Study [40], eligible patients with disease progression after first-line combination therapy with bevacizumab and a first- or second-generation TKI were enrolled. After disease progression, 71 of the 107 patients received tissue biopsies. The remaining 36 patients received liquid biopsies. In total, 59 patients were confirmed to have *T790M* mutations (55.1%), with 40/71 (56.3%) being identified by means of tissue re-biopsy and 17/30 (56.7%) through plasma testing, meaning that the two testing methods showed similar detection rates.

Several potential mechanisms for the development of drug resistance in late-stage EGFFR-mutant NSCLC patients after first- and second-line TKI treatment have been proposed. In addition to the acquired mutation of EGFR *T790M*, EGFR C797S mutation impacts the binding affinity of third-generation TKIs like osimertinib. JA et al. [41] observed that gefitinib-sensitive lung cancer cell lines developed resistance to gefitinib with focal amplification of the MET oncogene. In a German study, Wagener-Ryczek et al. [42] analyzed the spectrum of acquired resistance to various generations of TKIs. Participants were re-biopsied after 6 months of TKI treatment. In the erlotinib/gefitinib group, 56% developed *T790M* mutations, and 22% developed multiple mutations. In the afatinib group, 40% developed T790M mutations, and 11% developed multiple mutations. In a randomized, international, open-label, phase 3 trial, Mok et al. [43] recruited 419 advanced NSCLC patients with *T790M* resistance mutations. In a 2:1 ratio, the eligible patients were randomized to receive either osimertinib (80 mg, once daily) or systemic chemotherapy. The median duration of PFS was significantly longer in the osimertinib group than in the chemotherapy group (10.1 months vs. 4.4 months; hazard ratio: 0.30; 95% confidence interval [CI]: 0.23 to 0.41; *p* < 0.001). In the AURA study, osimertinib showed greater efficacy than chemotherapy in patients harboring *T790M* mutation, even in patients with brain metastasis.

Due to the significantly longer overall survival that was reported in the FLAURA trial, osimertinib has a dual role as a second- or first-line treatment of late-stage EGFR-mutated NSCLC. Different resistance incidences can develop following osimertinib administration due to the different lines of treatment for advanced EGFR-mutated NSCLC [44,45,46]. Resistance mechanisms can be categorized into acquired mutations, bypass signaling activation, and phenotypic transformation. Bypass signaling activation involves the upregulation of alternative pathways, such as *MET*, *HER2*, and *RAS-MAPK*, which allow tumor cells to sustain proliferation despite EGFR inhibition. Phenotypic transformation, including epithelial-mesenchymal transition (EMT) and small cell lung cancer (SCLC) transformation. In the AURA 3 study [43], sixty treatment-naive patients were randomized to receive either 80 mg (30 patients) once daily or 160 mg once daily. Forty-two (70%) patients experienced disease progression, and plasma NGS analyses were collected from 38 (91%) patients at or after disease progression. Of the AURA trial cohort of patients, 143 *T790M*-mutant NSCLC patients from four institutions received either a tumor or plasma genotyping assay before starting osimertinib treatment [44]. Forty-one patients experienced tumor progression and received tumor biopsies to carry out genomic NGS analysis of the tumor. Thirteen patients remained positive for EGFR *T790M* mutations (32%), and *C797S* mutations were detected in 32 patients (28%). A loss of *T790M* was observed in 28 patients (68%). The median time to osimertinib discontinuation (TTD) with persistent *T790M* mutation, identified by means of resistance biopsies, was 15.2 months, whereas the median TTD with lost T790M as per the resistance biopsy was 6.1 months.

To evaluate second-line osimertinib resistance, Le et al. [45] retrospectively surveyed The University of Texas MD Anderson Lung Cancer Moon Shot GEMINI database for advanced NSCLC patients who had been treated with osimertinib and the Moffitt electronic health record, Clinical Genomic Action Committee database, from Moffitt Cancer Center and Research Institute (MCC) for NSCLC patients with EGFR T790M mutations. At MDACC, a 50-gene panel was performed for tissue samples and digital-droplet PCR was used for blood samples. Pyrosequencing of the EGFR gene and Moffitt Illumina TruSight Tumor 26 (TST26) sequencing were performed at the MCC. Commercially available NGS platforms, e.g., FoundationOne and Guardant360, were used at both sites. A total of 118 patients were enrolled. The median time to treatment failure (TTF) based on prior EGFR TKI treatment was 14 months. The median follow-up on osimertinib was 13 months. The subgroup analysis showed that pre-existing CNS metastasis led to worse outcomes.

Papadimitrakopoulou et al. [46] conducted a plasma ctDNA analysis of enrolled patients in the AURA3 study. Co-mutation, along with *T790M* mutation, was detected, including *TP53* (64%) and EGFR amplification (33%). Since the FDA granted osimertinib approval as a first-line treatment in April 2018 based on the significant PFS benefit demonstrated in the FLAURA trials, first-line resistance to osimertinib has been under study. Yu et al. [47] conducted the ORCHARD study, an open-label, multicenter, multi-drug, biomarker-directed, phase 2 platform study, enrolling eligible late-stage EGFR-mutant patients with disease progression following first-line osimertinib monotherapy. Using the longitudinal samples, a ctDNA analysis of the tumor burden was carried out. Thirteen combination arms were arranged in the platform study to evaluate the efficacy, safety, and tolerability of combination treatment, and the study is estimated to be completed in June 2025. Chmieleck et al. [48] reported a study on first-line osimertinib resistance based on the FLAURA study. A total of 279 patients were randomized to an osimertinib group, and an acquired resistance analysis was conducted in 109/137 of the patients. The investigators found that 38/109 (35%) of the patients had a detectable mechanism of resistance. The most common acquired resistance mechanism was *MET* amplification (16%), followed by mutations in EGFR (10%). *C797S* mutation occurred in seven patients (6%), and *L718Q* mutation occurred in two patients.

Tamiya et al. [49] conducted a prospective observational study in Japan, the ELUCIDATOR study, to survey the acquired resistance to first-line osimertinib treatment. Serial plasma samples were collected from eligible enrolled patients at baseline, at 3 months, at 12 months, and after the detection of disease progression. Plasma samples from 178 patients were analyzed using NGS. The PFS of the patients harboring L858R was significantly lower than that of patients carrying EGFR *Exon 19* deletions (median PFS: 17.2 [95% CI: 12.0–19.5] months vs. 23.3 [95% CI: 14.5–30.2]). The PFS and OS rates of the patients carrying *TP53* co-mutations in ctDNA were significantly lower than those of the patients without *TP53* mutations in ctDNA (median PFS: 12.5 [95% CI: 7.1–19.3]). The PFS and OS rates of the patients carrying EGFR amplification in ctDNA were significantly lower than those of the patients without EGFR amplification in ctDNA. The PFS and OS rates of the patients carrying *MET* amplification in ctDNA were significantly lower than those of the patients without *MET* amplification in ctDNA. The patients carrying *PIK3CA* co-mutations had lower PFS rates than the patients without *PIK3CA* mutations. A. Leonetti et al. [50] conducted a single-center prospective study in Italy, recruiting TKI-naive late-stage EGFR-mutant patients or patients experiencing disease progression after first-line EGFR-TKIs. Sixty-five patients were prospectively enrolled, and the median follow-up time was 25.2 months. NGS analyses of tissue samples were performed at baseline and disease progression. *TP53* was the most frequent co-mutation (59.7%), followed by *CDK4* (14.5%) and *TERT* (12.9%). The patients carrying *TP53* co-mutations had significantly lower PFS rates (12.2 months vs. not reached; *p* = 0.017) and shorter median durations of response (DoRs) (9.8 months vs. not reached; p = 0.007). Thirty-five (56.5%) patients developed disease progression, and sixteen patients underwent tissue re-biopsies. In addition, 26/29 (89.7%) of the patients underwent liquid biopsies. EGFR-dependent resistance mechanisms were observed, including EGFR *C797S* (*n* = 3), *L718Q* (*n* = 1), *T751I* (*n* = 1), Exon 20 insertion (*n* = 1) mutation, and EGFR amplification (*n* = 6). For the concordance analysis between tissue and plasma at PD, the NGS panels of the tissue and plasma of 16/29 of the patients were analyzed, and a high mean concordance rate (14/16, 87.5%) was noted.

In the phase 3 FLAURA study, the most common resistance mechanism was *MET* amplification (16%), followed by EGFR C797S mutation (6%). Potential early-onset acquired resistance mechanisms were identified based on plasma samples that were collected at disease progression and/or treatment discontinuation in the FLAURA study. In the patient group with osimertinib administration as the first-line treatment, 10–25% developed EGFR-dependent mutations [51], and the most common cause of EGFR-dependent mutation was EGFR *Exon 20 C797S* mutations leading to the disruption of the *cysteine 797* binding site [52,53,54,55,56]. Miscellaneous EGFR mutations, such as *L792 H/L792 V*, *G796S/G796C*, *G724S*, and *G719A*, have been associated with osimertinib resistance [52,56]. Minari et al. [56] conducted a review article discussing the mechanisms of resistance to third-generation EGFR-TKIs. EGFR-dependent mechanisms of resistance include C797S mutation, p.L798I, p.L692V and p.E709K. EGFR-independent mechanisms of resistance include bypass pathway activation, HER2 and MET amplification, PIK3CA activating mutations PTEN deletion, RAS-MAPK pathway activation. Phenotypic alterations in the mechanisms of resistance include small cell lung cancer (SCLC) transformation.

Yang et al. [57] conducted a study, using NGS for 416 cancer-relevant geneson 93 osimertinib-resistant lung cancer patients’ samples. EGFR *G796/C797*, *L792*, and *L718/G719* mutations were identified in 24.7%, 10.8%, and 9.7% of the cases, respectively. In the Phase III FLAURA study, Chmielecki et al. [58] identifies acquired resistance mechanisms to first-line osimertinib with NGS assessing circulating-tumor DNA from paired plasma samples (baseline and disease progression/treatment discontinuation) in patients with baseline EGFR mutation. The most frequent resistance mechanisms to first-line osimertinib are MET amplification (*n* = 17;16%) and EGFR C797S mutations (*n* = 7; 6%); In contrast, as shown in the previous studies, the most frequent resistance mechanisms to second-line osimertinib treatment, are acquired EGFR mutations (e.g., C797S), and amplification of MET and ERBB2 (HER2). In the FLAURA study, 279 patients were randomized to osimertinib and 277 patients to comparator EGFR-TKI. Chmielecki et al. studied the acquired resistance mechanisms by treatment arm with plasma ctDNA analysis. In the osimertinib arm, the most common acquired resistance mechanism detected was *MET* amplification, occurring in 17 patients (16%), followed by mutations in EGFR in 11 patients (10%). In the comparator EGFR-TKI arm, the most common acquired resistance mechanism detected was EGFR *T790M* mutation, followed by *MET* amplification (6%) and *CDK6* amplification (4%). In the FLAURA study [22,23], paired plasma samples were acquired from 109 patients and analyzed using NGS before treatment and after disease progression. The most common resistance after first-line treatment with osimertinib was *MET* amplification (16%), followed by EGFR *C797S* mutation (6%). In the comparator group (first-line TKI treatment with gefitinib or erlotinib), the most common resistance mechanism was *T790M* mutation (44%), followed by *MET* amplification (6%). Besides EGFR mutations, NGS analyses of tissues from patients in both groups showed additional mutations, including *TP53* (62%), EGFR amplification (20%), *RB1*, *RBM10*, *HER2*, *MET*, *SMARCA4*, and *RICTOR*. In terms of second-line treatment with osimertinib for advanced-stage NSCLC patients, in the AURA study [43], the most common resistance mechanisms were *MET* amplification and EGFR *C797S* mutation. About half of the patients exhibited a loss of detectable EGFR *T790M*.

In an Italian study, Leonetti et al. [53] enrolled advanced EGFR-mutated NSCLC patients who were going through first- or second-line treatment with osimertinib from May 2018 to November 2022. Sixty-two patients received NGS of their tissue or a corresponding plasma sample at baseline. On disease progression, paired tissue biopsies and plasma sample tests were performed. Overall, *MET* amplification was the most frequent resistance mechanism (24%), followed by *MET* amp + EGFR *C797S* (8%). In the concordance analysis of NGS based on tissue or corresponding plasma specimens to assess the disease progression, the mean concordance rate was 87.5%. In Taiwan, Liao et al. [59] conducted a real-world cohort study, working with the Taiwan Cooperative Oncology Group, and performed T1521 NGS analysis of patients with advanced NSCLC. In cohort 1, an EGFR-mutated and pretreated group, 11.6% (28/250) had *MET* amplification, and 4.0% (10/250) had *ERBB2* amplification. In patients who were treated with osimertinib, EGFR *C797S* (6.2%, 5/81) and BRAF V600E (2.5%, 2/81) mutations were detected.

EGFR-independent pathways of drug resistance have been studied. An epithelial-to-mesenchymal transition (EMT) can be the driver of acquired resistance to EGFR TKIs, as can *AXL* tyrosine kinase [60,61], the activation of *Notch-1* [62,63], the activation of Insulin-like growth factor 1 receptor (*IGF1R*) [64,65], *MAPK*, and the *AKTSrc/FAK* network [66], which converges on *AKT* and *MAPK* to sustain oncogenic signaling and induce EMT.

## 3. Co-Mutations with Oncogenes

Late-stage adenocarcinoma patients with EGFR mutations may harbor other mutated protooncogenes or dysregulated tumor suppressor genes. In previous review studies [45,51], common concurrent genetic mutations included *TP 53* (55–65%), *MET* alterations (polysomy: 23–29%; amplification: 3%), and *PIK3CA* mutations (3.5%). Qin et al. [67] conducted a meta-analysis of concurrent *TP53* mutations in patients with EGFR or anaplastic lymphoma kinase (*ALK*) mutations. In the final analysis, 15 studies involving 1342 patients were included and showed that concurrent *TP53* mutation was associated with unfavorable PFS (HR = 1.88; 95%CI: 1.59–2.23; *p* < 0.001) and OS (HR = 1.92; 95%CI: 1.55–2.38; *p* < 0.001). In their meta-analysis of the prognostic impact of EGFR-*TP53* co-mutations, Ferrara et al. [68] found that the TP53 co-mutant group had shorter PFS versus the EGFR-mutant/*TP53* wild-type group (HR = 1.67; 95% CI 1.51–1.83; *p* = 0.18).

To evaluate the impact of concurrent *PIK3CA* mutations on the response to EGFR-TKIs, Juliana Eng et al. [69] retrospectively identified relevant patients at the New York Memorial Sloan Kettering Cancer Center between 2009 and 2013. Thirty-seven patients had a concurrent *PIK3CA* mutation. For EGFR-mutant lung adenocarcinoma patients, OS was shorter than for patients harboring a single EGFR mutation (95% CI: 28.2 to NA months; *p* = 0.006). In the *PI3K/Akt* networks, three classes of *PI3Ks* have been identified [70]. Class I *PI3Ks* play a crucial role in the development of cancers. The activation of the *PI3K/Akt* signaling pathway involves various factors, including the RTK family, Toll-like receptors (TLRs), and B-cell antigen receptors (BCRs). As a downstream effector on serine and threonine, *mTOR* is reported to regulate tumor growth, survival, metabolism, and immunity. The *PI3K/Akt* signaling pathway is important for the drug resistance of various types of cancer, including lung cancer. *PI3K/Akt* inhibitors can inhibit tumor growth and further induce the apoptosis of tumor cells. The *PI3K* pathway promotes metastasis by reducing intercellular adhesion and enhancing mobility. Daher et al. [71] conducted a study with a large dataset of NSCLC patients with *PIK3CA* mutations. When comparing patients with single *PIK3CA* mutations to patients with concurrent gene alteration, the latter group was younger, and there were more female patients with adenocarcinoma in their pathology. *PI3K/Akt* pathway dysregulation also plays an important role in cancer drug resistance [72]. *PIK3CA* mutations can trigger *AKT* and *mTOR* in the *AKT/mTOR* pathway, further promoting the activation of bypass pathways, downstream pathway activation, epithelial-to-mesenchymal transitions (EMTs), and cell survival and proliferation.

*MET* is a disulfide-linked heterodimeric RTK with an extracellular αchain and β chain. The intracellular component includes a juxta-membrane region that is responsible for signal downregulation and receptor degradation. The C-terminal region can act as a docking site, leading to downstream signaling cascades, such as phosphoinositide 3-kinase (*PI3K*), signal transducers and activators of transcription proteins (*STATs*), and mitogen-activated protein kinase (*MAPK*).

*MET* amplification is caused by an increase in the *MET* gene copy number and can cause successive protein overexpression and tyrosine kinase activity. It can function as a mechanism of resistance after EGFR-TKI treatment [73,74,75,76,77]. Three methods can be used to detect MET amplifications: fluorescence in situ hybridization (FISH), quantitative real-time polymerase chain reaction, and Next Generation Sequencing (NGS). The gold standard for the detection of *MET* amplification is fluorescent in situ hybridization (FISH) from solid tissue biopsies [78]. NGS is an alternative to FISH for the detection of *MET* alterations, such as *Exon 14* skipping mutations or amplification. DNA- or RNA-based NGS can be used to diagnose *MET Exon 14* skipping mutations. FISH is a widely accepted standard due to its strong correlation with treatment outcomes [79]. Camidge et al. [79] enrolled 38 patients with a *MET*-to-CEP7 ratio above or equal to 1.8 based on FISH results and conducted a stratified analysis according to the patients’ MET amplification levels with oral administration of crizotinib. The patients with higher MET amplification levels had higher objective response rates. MET Exon 14 skipping mutations occurred in 3 to 4%, and *MET* amplifications occurred in 1 to 6% of NSCLC patients. In the GEOMETRY mono-1 trial, Wolf et al. [80] conducted a multiple-cohort, phase 2 study and enrolled advanced NSCLC patients with MET Exon 14 skipping mutation or MET amplification. They found that 97 patients had MET Exon 14 skipping mutation, and 210 had MET amplification. The patients who had been previously treated were enrolled in cohorts 1 through 4. An overall response, as assessed by an independent review committee, was observed in 41% of the 69 previously treated patients and in 68% of the 28 treatment-naive patients. The median duration of response was 9.7 months among previously treated patients and 12.6 months among treatment-naive patients. Paik et al. [81] conducted an open-label, phase 2 study with the administration of tepotinib (at a dose of 500 mg) once daily for eligible advanced-stage NSCLC patients with a confirmed MET Exon 14 skipping mutation. The testing of MET Exon 14 skipping mutations was performed centrally on circulating free DNA (cfDNA) obtained from plasma (liquid biopsy), with the use of the NGS panel Guardant360 or by evaluating RNA that had been obtained from fresh or archival (formalin-fixed, paraffin-embedded) tumor biopsy tissue with the use of the Oncomine Focus Assay. The response rate was 46% (95% confidence interval [CI]: 36 to 57), as determined by means of an independent review, and the median duration of response was 11.1 months in the combined biopsy group.

MET is a proto-oncogene located on chromosome 7q21–q31 that encodes for a high-affinity transmembrane receptor tyrosine kinase (RTK). The MET receptor is composed of an extracellular region, and immunoglobulin domains; and an intracellular region with a juxta-membrane domain, catalytic tyrosine kinase domain, and carboxy-terminal docking site. With the certain binding of its ligand, activates its intracellular tyrosine kinase catalytic activity, leading to activation of downstream RAS/ERK/MAPK, PI3K/AKT, Wnt/β-catenin, and STAT signaling pathways. Such cascades are associated with cell proliferation, survival, migration, and play an important role in embryonic development, wound healing, and tissue regeneration [82].

Amivantamab, a human monoclonal antibody that is derived from an IgG1 isotype, is able to bind to receptors of Fc fragments of IgG (FcγRs) on immune cells [83,84], binds the extracellular domains of EGFR and MET and blocks the ligand in a dose-dependent manner. Amivantamab also induces apoptosis and antibody-dependent cellular cytotoxicity (ADCC), a mechanism of cell-mediated immune defense whereby an effector cell, which is classically known to be the natural killer (NK) cell of the immune system, kills a target cell, whose membrane-surface antigens have been bound by specific antibodies and independent of the immune complement system. In addition to the approval for the standard treatment for *Ex20ins*-mutant NSCLC, amivantamab–lazertinib treatment has shown efficacy and safety as a first-line treatment of advanced EGFR-mutant NSCLC compared with osimertinib monotherapy in the MARIPOSA trial [32]. The treatment efficacy of amivantamab–lazertinib is not affected by secondary mutations of the tyrosine kinase domain of EGFR, such as C797S, L718Q, G742S, and S769I. In a secondary analysis based on MARIPOSA, Felip et al. [84] showed improvement of the median PFS (mPFS) in the amivantamab–lazertinib group compared with osimertinib for patients with TP53 co-mutations (18.2 versus 12.9 months; HR: 0.65 [95% confidence interval (CI): 0.48–0.87]); meanwhile, patients in the amivantamab–lazertinib group showed prolonged mPFS compared with the osimertinib group among randomized patients with baseline liver metastases [18.2 versus 11.0 months; HR: 0.58 (95% CI: 0.37–0.91); *p* = 0.017] and without baseline liver metastases [24.0 versus 18.3 months; HR: 0.74 (95% CI: 0.60–0.91); *p* = 0.004].

## 4. NGS and Associated Concordance

NGS has become a useful tool in clinical practice for monitoring resistance mutations, guiding subsequent treatment decisions, and tracking tumor heterogeneity. It can be performed using tissue biopsy or liquid biopsy (circulating free DNA, cfDNA) to detect key resistance-associated alterations, such as EGFR *T790M*, *C797S*, *MET* amplification, *HER2* amplification, and *PIK3CA* mutations. Liquid biopsy facilitates the real-time, non-invasive monitoring of treatment response and early detection of emerging resistance mechanisms, enabling timely therapeutic adjustments. International oncology societies have recommended the use of liquid biopsy with NGS for the molecular assessment of lung cancer [51]. Circulating tumor DNA (ctDNA) can be implemented as a tool for early diagnosis, molecular analysis, treatment decisions, and response monitoring [85,86]. To test the efficiency of pretreatment liquid NGS, Yang et al. [87] enrolled 180 patients with suspected advanced NSCLC. All participants received cfDNA-NGS using the Guardant360 74 gene assay (Guardant Health, Redwood City, CA, USA) after signing informed consent forms and being randomized into two groups. In one group, the physicians started treatment after receiving both a tissue and liquid biopsy report. In the other group, the treating physicians started treatment after receiving a liquid biopsy report. In the liquid biopsy-only group, the patients had a shorter waiting period before starting targeted therapy. A high concordance rate was noted in the patient group with identified driver mutations.

In a Spanish study, Arriola et al. [88] enrolled 154 eligible patients with paired tumor and serum samples. The concordance rate for EGFR mutation status between the tissue and plasma samples was 88.8%, with a sensitivity of 45.5%, and the specificity was 96.7%. van der Wel et al. [89] enrolled eligible advanced stage NSCLC patients that progressed on the second or the third line osimertinib treatment. Plasma sequencing used AVENIO Expanded Panel and tumor biopsies with DNA and RNA sequencing were performed. The driver mutation was detected in 42/51 plasma samples (82%) and in 50/51 tumor samples (98%), concordance rate was 80%. The results suggest that paired plasma and tumor samples can identify resistance mechanisms in the advanced stage NSCLC patients that progressed on the second or the third line osimertinib treatment with potential for exploration of tailored and precision treatment. In a European prospective, single-center study [90], 50 metastatic NSCLC patients with disease progression after second-line therapy with osimertinib were enrolled, received tumor tissue biopsies, and provided blood samples for ctDNA analysis. DNA and RNA were isolated from the tissue; DNA NGS was performed using the Illumina AmpliseqTM Cancer Hotspot Panel v2-SOCv1 (Illumina, Inc., San Diego, CA, USA), and the RNA NGS was carried out using the Archer FusionPlex Lung version 1 (IDTDNA, previously Invitae and Archer Dx, Boulder, CO, USA). For the ctDNA analysis, variants were reported when the variant allele frequency (VAF) was at least 0.10%. Forty-one patients provided matched samples with successful molecular profiling in the tissue and blood ctDNA analysis. The concordance rate of the primary driver identification was 80.4%.

To improve the success rate of NGS panel testing and shorten the turnaround time, a Japanese study [90] adopted the AMOY 9-in-1 kit. The AMOY kit is capable of studying known targetable driver mutations or fusions of nine genes (EGFR, ALK fusion, ROS1 fusion, RET fusion, MET Ex.14 skipping mutations, HER2, BRAF V600E mutation, KRAS, and NTRK fusion) by means of real-time polymerase chain reaction (PCR). In the study, the success rates of AMOY and NGS were 98.5% and 87.8%, respectively. A shorter turnaround time was noted in the AMOY group.

## 5. Treatment Strategy and Further Management After Drug Resistance

The NCCN has approved osimertinib and chemotherapy combination treatment and amivantamab plus lazertinib treatment as first-line treatments for advanced NSCLC patients harboring EGFR mutations. Drug resistance after various first-line regimens may evolve with different resistance presentations. Several clinical trials and preclinical studies support the efficacy of combination therapies in overcoming resistance in late-stage EGFR-mutant NSCLC. For MET-driven resistance, combination therapies involving osimertinib and MET inhibitors (e.g., savolitinib, capmatinib, tepotinib) have demonstrated promising results. The TATTON study [91] is a multi-arm, multicenter, open-label phase Ib study divided into several parts. In Part A, osimertinib 80 mg was administered with savolitinib 600 mg in the initial dose, and then 800 mg once daily was administered. In Part B, the group was subdivided into three groups. The B1 group consisted of eligible patients with previous third-generation EHGR-TKI treatment. The B2 group was the eligible T790M-negative patients with no prior third-generation TKI treatment. The B3 group was the eligible T790M-positive patients with no prior third-generation TKI treatment. As for the Part D group, eligible T790M-negative patients with no prior third-generation TKI treatment were administered osimertinib 80 mg per day and savolitinib 300 mg once daily.

With the lower dose of savolitinib in combination with osimertinib the slight improvement of the tolerability was observed. The TATTON trial and the SAVANNAH trial showed that combining osimertinib with MET inhibitors improved response rates in patients with MET amplification.

For bypass pathway activation, EGFR TKI + MEK inhibitors (e.g., osimertinib + selumetinib) have been explored to counteract RAS-MAPK activation. Similarly, EGFR TKI + anti-angiogenic agents (e.g., osimertinib + bevacizumab) have been investigated to suppress tumor progression. In the phase 2 WJOG9717L study [92], untreated patients with non-squamous NSCLC-harboring EGFR mutations were randomized to receive daily osimertinib plus bevacizumab (15 mg/kg every 3 wk) or osimertinib alone. In one Chinese meta-analysis [93], 824 eligible patients were included in 10 randomized controlled trials. The experiment group (osimertinib plus bevacizumab) had a higher ORR (relative risk [RR] = 1.23, 95% confidence interval [CI] = 1.03–1.47, *p* = 0.02). A significant reduction in the level of CEA and VEGF was noted in the experiment group. In the West Japan Oncology Group 8715L phase 2, randomized clinical trial [94], Akamatsu et al. recruited advanced lung adenocarcinoma patients who had disease progression with prior EGFR-TKI treatment and randomized the eligible patients to osimertinib plus bevacizumab or osimertinib alone in a 1:1 ratio. The combination therapy group did not show PFS prolongation compared with the osimertinib monotherapy group.

In the European Thoracic Oncology Platform (ETOP 10–16) BOOSTER trial [95], 155 eligible patients were enrolled from six European countries and randomized to osimertinib plus bevacizumab or osimertinib alone. Similarly, there was no significant difference in median PFS between the combination group and the osimertinib alone group. In the subgroup analysis, current and former smokers had a significantly longer PFS with combination treatment [HR: 0.57 (0.33–0.98); Wald test *p* = 0.043). In Europe, EGFR mutations are found in 12–15% of the NSCLC patient population [96]. A Swiss retrospective cohort study enrolled 149 eligible patients with EGFR-mutated NSCLC between November 2015 and May 2022 [97]. Seventy-eight patients experienced disease progression. In addition to local ablative treatment, 40 patients received second-line treatment, including 18 patients receiving carboplatin/pemetrexed 18 (45.0%), eight patients receiving carboplatin/paclitaxel/bevacizumab/atezolizumab (IMPOWER 150) (20.0%), three patients receiving carboplatin/paclitaxel 3 (7.5%), and three patients receiving afatinib 3 (7.5%). Some of the remaining eight patients (20.0%) received off-label MET inhibitors such as savolitinib and tepotinib. The OS was 51.6 months in this real-world Swiss cohort study, and the OS of the patients whose illness developed into oligo-progressive disease and who received local ablative treatment was 60 months. This surprising result underscores the importance of frequent surveys and timely specified locoregional treatment according to the personalized treatment approach. In an Asian retrospective study [98], 60 eligible patients were included. The initial first-line treatment was osimertinib monotherapy. The median time to treatment failure was 14.4 months. The median time to initiation of the second-line treatment of pemetrexed–platinum chemotherapy plus osimertinib was 41 days. The median time to treatment failure of the second-line treatment with pemetrexed–platinum chemotherapy plus osimertinib was 6.6 months.

Several immunotherapy–chemotherapy combination trials have been carried out, including Checkmate-722 [99], KEYNOTE-789 [100], ORIENT-31 [101], IMpower150 [102], ATTLAS [103], and IMpower151 [104]. The phase 3 CheckMate 722 trial enrolled 294 eligible patients with disease progression after first- or second-generation EGFR TKI therapy without EGFR T790M mutations or osimertinib with or without T790M mutations. Eligible patients were randomly assigned at a ratio of 1:1 to nivolumab (360 mg once every 3 weeks) plus platinum doublet chemotherapy (once every 3 weeks) or platinum doublet chemotherapy alone (once every 3 weeks) for four cycles. After a median follow-up of 38.1 months, PFS was not significantly improved following nivolumab plus chemotherapy versus chemotherapy (median: 5.6 vs. 5.4 months; hazard ratio [HR]: 0.75 [95%CI: 0.56 to 1.00]; *p* = 0.0528). In the phase 3 KEYNOTE-789 study [100], 489 stage IV non-squamous NSCLC with common EGFR mutations and progression after EGFR-TKI treatment were enrolled and randomly assigned at a ratio of 1:1 to 35 cycles of pembrolizumab (200 mg) or placebo once every 3 weeks, plus four cycles of pemetrexed and carboplatin or cisplatin once every 3 weeks and then maintenance pemetrexed. The primary end points were the PFS and OS rates of the eligible patients. Statistically, the PFS and OS rates of patients with EGFR-mutant, TKI-resistant metastatic non-squamous NSCLC did not increase significantly in the pembrolizumab plus chemotherapy group compared with the PFS and OS rates of the chemotherapy group. In the subgroup analysis, the OS in the pembrolizumab plus chemotherapy group at 24 months was 37.7%, whereas the OS in the chemotherapy group was 26.0%. This may imply the potential treatment benefit through careful selection of the eligible EGFR-mutant NSCLC patients with PDL1 TPS ≥ 1%. In the Asian phase 3 ORIENT-31 trial, 476 eligible patients were enrolled and randomly assigned in a 1:1:1 fashion (158 to the sintilimab plus IBI305 plus chemotherapy group, 158 to the sintilimab plus chemotherapy group, and 160 to the chemotherapy alone group). The treatment group was administered sintilimab plus bevacizumab biosimilar IBI305 plus chemotherapy (pemetrexed and cisplatin). A significant PFS benefit was noted with sintilimab plus IBI305 plus chemotherapy compared with chemotherapy alone (median: 7.2 months [95% CI: 6.6–9.3]; HR: 0.51 [0.39–0.67]; two-sided *p* < 0.0001). No OS benefit was noted in the sintilimab plus IBI305 plus chemotherapy group. In the forest plots of a meta-analysis [105], the analyzed hazard ratios for PFS, including those from the KEYNOTE-789 [HR: 0.80 (0.65–0.97)], ORIENT-31 [HR: 0.72 (0.55–0.94)], and ATTLAS [HR: 0.62 (0.45–0.86)] trials, favored immunotherapy–chemotherapy combination treatment. However, the hazard ratios for OS did not indicate a significant advantage of immunotherapy–chemotherapy combination treatment.

## 6. Future Perspectives

**Figure 1:**
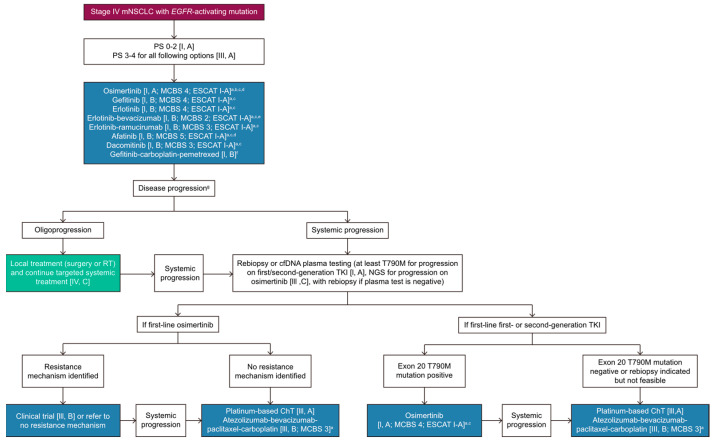
Treatment algorithm for stage IV EGFR-mutated NSCLC according to the 2023 ESMO Clinical Practice Guideline (adapted from [109]).

EGFR-mutated NSCLC patients constitute nearly half of all lung cancer patients in the Asian population. The initial treatment selection may depend on the pretreatment NGS results and initial tumor burden, as well as the prognosis regarding possible co-mutation. In the TRACER X study, Abbosh et al. [106] followed 197 early-stage lung cancer patients for 5 years after surgery using ctDNA. ctDNA was detected postoperatively (before or after relapse) in 59 out of 70 (84%) of these patients, suggesting that liquid biopsy surveillance can be a useful tool for detecting relapse. In the Korean Lung Cancer Consortium (KLCC-12-02), Lee et al. [107] conducted a study where the longitudinal monitoring of plasma EGFR mutations was performed in 81 NSCLC patients who had been treated with EGFR TKIs. Emerging resistance was observed based on the early detection of T790M as a secondary mutation in 14 (28.6%) of the 49 patients. In one study [108], 50 advanced NSCLC patients were enrolled, and their cfDNA was collected from sputum, plasma, urine, and tumor tissue. The somatic genomic alterations in the liquid biopsies were registered and measured using the same next-generation sequencing (NGS) platform. The overall concordance rates were 86% in plasma cfDNA, 74% in sputum cfDNA, and 70% in urine cfDNA. This result implied a potential use of sputum as a surrogate for the tracing and surveillance of tumor relapses. The ESMO clinical practice guideline for oncogene-oriented NSCLC [105,109] states that adequate tissue should be obtained for standard histological diagnosis and molecular testing. Molecular testing should include EGFR mutation, ALK translocation, ROS1 translocation, RET translocation, *BRAF V600* mutation, *NTRK* translocation, *HER2* mutation, EGFR *ex20ins* mutation, and *MET Exon 14* skipping mutation (Figure 1). In the treatment approach for patients with advanced metastatic EGFR-mutated NSCLC [110], several aspects should be considered and anticipated, including the tumor burden, impact of co-mutation, acquired resistance to the prior treatment, and subsequent treatment strategies at progression. ASCO also published its living guideline as a reference for therapy for Stage IV NSCLC with driver alterations in February 2024 [111], although the NCCN updated its guideline in October 2024 with a newly FDA-approved regimen. For the management of drug resistance after TKI treatment, several clinical trials of fourth-generation EGFR TKIs are currently being carried out [112] (Table 2).

Among these trials, the most promising fourth-generation TKI is the BLU945. The BLU945 [113,114], a potent, reversible, wild-type-sparing inhibitor of EGFR+/T790M and EGFR+/*T790M/C797S* resistance mutants, showed promising results in the mouse models. In the osimertinib-resistant mouse xenograft models, BLU945 demonstrated significant tumor growth inhibition. BLU-945 also demonstrated tumor volume decreases in patients from the SYMPHONY trial [114]. To determine the effectiveness of antibody–drug conjugates, several phase 2 and phase 3 trials involving the post-progression EGFR-mutant NSCLC patients are ongoing. In 2019, Scharpen-seel et al. [115] analyzed 148 NSCLC tissues, and highly positive *HER3* staining was observed in 82.7% of the primary NSCLC tumors. The *HER3*-directed antibody–drug conjugate patritumab deruxtecan (*HER3*-DXd) is an investigational antibody–drug conjugate (ADC) composed of a fully human anti-*HER3* immunoglobulin G1 monoclonal antibody, patritumab. *HER3*-DXd is covalently linked to topoisomerase I inhibitor payload via a tetrapeptide-based, tumor-selective, stable, and cleavable linker. In the phase 2 HERTHENA-Lung01 trial [116], *HER3*-DXd demonstrated evidence of efficacy with durable responses. The phase 3 HERTHENA-Lung02 trial [117] for evaluating HER3-DXd in patients with EGFR-mutated NSCLC after progression during treatment with an EGFR TKI and cisplatin-based chemotherapy is ongoing. In September 2024, the phase 3 HERTHENA-Lung02 trial showed a statistically significant improvement in progression-free survival (PFS) compared with platinum-based chemotherapy, although the evaluation of OS was premature at the time of this analysis. No new safety signals were reported in the HER3-DXd group, except for several reports of interstitial lung disease (ILD) events. In the phase 3 TROPION-Lung01 study [118], Dato-DXd significantly improved PFS compared with docetaxel; no OS benefit was noted in this study.

To summarize, pretreatment NGS and evaluating the initial tumor burden and intrinsic co-mutation are reasonable options when starting EGFR-TKI or combination treatment for late-stage EGFR-mutant NSCLC patients. After starting EGFR TKI treatment, regular monitoring of changes in the disease may help detect early disease progression. Serum or even sputum NGS may be promising surrogates if a re-biopsy cannot be performed successfully. Post-progression treatment modality studies are ongoing, and re-biopsy NGS research can provide clinicians with a new road map for developing personalized and tailored treatments for patients. Locoregional treatment modalities can be utilized in the pursuit of longer OS. New-generation TKIs and antibody–drug conjugate (ADC) trials are ongoing [119] (Table 3) and may open new horizons for the treatment of post-progression EGFR-mutated NSCLC patients.

## Figures and Tables

**Table 1:** FDA-approved EGFR-TKI, TKI/CT, and TKI treatments and associated pivotal trials.

TKI	Study (Reference)	Design	Phase	PFS (Months)	OS (Months)	ORR (%)
Gefitinib	IPASS [1,2]	Gefitinib vs. carboplatin plus paclitaxel	3	9.5 vs. 6.3	21.6 vs. 21.9	EGFR mutation: 72.1 vs. 47.3
	NEJ002 [10,11]	Gefitinib vs. carboplatin plus paclitaxel	3	10.8 vs. 5.4	27.7 vs. 26.6	73.7 vs. 30.7
	WJTOG 3405 [12]	Gefitinib vs. cisplatin plus docetaxel	3	9.2 vs. 6.3	34.8 vs. 37.3	62.1 vs. 32.2
Erlotinib	EURTAC [13]	Erlotinib vs. platinum-based doublets	3	9.7 vs. 5.2	22.9 vs. 19.6	58 vs. 15
	OPTIMAL [14]	Erlotinib vs. carboplatin plus gemcitabine	3	13.1 vs. 4.6	22.8 vs. 27.2	83 vs. 36
Afatinib	Lux-Lung3 [16,18]	Afatinib vs. cisplatin plus pemetrexed	3	11.1 vs. 6.9	28.2 vs. 28.2	56 vs. 23
	Lux-Lung6 [17,18]	Afatinib vs. cisplatin plus gemcitabine	3	11 vs. 5.6	23.1 vs. 23.5	66.9 vs. 23
Dacomitinib	ARCHER 1050 [20]	Dacomitinib vs. gefitinib	3	14.7 vs. 9.2	34.1 vs. 26.8	75 vs. 72
Osimertinib	FLAURA [22,23]	Osimertinib vs. gefitinib or erlotinib	3	18.9 vs. 10.2	NR	80 vs. 76
Osimertinib plus Chemotherapy	FLAURA 2 [24]	Osimertinib plus chemotherapy vs. osimtertinib alone	3	29.4 vs. 19.9	NR	83 vs. 76
Amivantamab	MARIPOSA [32]	amivantamab–lazertinib vs. osimtertinib alone	3	23.7 vs. 16.6	NR	86 vs. 85

**Table 2:** Fourth-generation EGFR inhibitors in clinical trials (adapted from [112]).

Drug	Sponsor	Activating Mutations (Preclinical)	Resistance Mutations (Preclinical)	Clinical Trial	Phase	Enrollment	Study Intervention	Recruitment Status
BLU-701	Blueprint Medicines	*cEGFR* *	*cEGFR*/C797S *cEGFR*/T790M/C797S	NCT05153408 (HARMONY)	1/2	20	BLU-701 BLU-701 + osimertinib BLU-701 + PBC	Terminated by the Sponsor
BBT-176	Bridge Biotherapeutics	*cEGFR*	*EGFR* ex19del/T790M *cEGFR*/C797S *cEGFR*/T790M/C797S	NCT04820023	1/2	45	BBT-176	Terminated by the Sponsor
BLU-945 (tigozertinib)	Blueprint Medicines	EGFR L858R	*cEGFR*/T790M EGFRL858R/C797S *cEGFR*/T790M/C797S	NCT04862780 (SYMPHONY)	1/2	190	BLU-945 BLU-945 + osimertinib	Terminated by the sponsor
TRX-221	Therapex Co., Ltd.	*cEGFR*	*cEGFR*/C797S *cEGFR*/T790M/C797S	NCT06186076	1/2	115	TRX-221	Not yet recruiting
BP1-361175	Xcovery Holdings, Inc.	*cEGFR*	*cEGFR*/C797S EGFR ex19del/ T790M/C797S	NCT05393466	1/2	30	BPI-361175	Recruiting
BDTX-1535	Black Diamond Therapeutics	*cEGFR*	*cEGFR*/C797S *cEGFR*/T790M/C797S	NCT05256290	1/2	200	BDTX-1535	Recruiting
JIN-A02	J Ints Bio	*cEGFR*	*cEGFR*/T790M EGFR ex19del/C797S *cEGFR*/T790M/C797S	NCT05394831	1/2	150	JIN-A02	Recruiting
BAY 2927088	Bayer	*cEGFR*	*cEGFR*/C797S	NCT05099172	1	460	BAY 2927088	Recruiting
H002	R&G Pharma Studies Co., Ltd.	*cEGFR*	*cEGFR*/T790M; *cEGFR*/C797S; *cEGFR*/T790M/C797S	NCT05552781	1/2	76	H002	Recruiting

cEGFRs *: common EGFR-activating mutations (Exon 19 deletions, Exon 21 L858R point mutations); EGFR: epidermal growth factor receptor.

**Table 3:** Major ADC and chemoimmunotherapy trials with EGFR-mutated NSCLC patients following progression on 3rd-generation EGFR-TKIs (adapted from [119]).

Study Name	Phase	Intervention Arm	Comparator Arm	Biomarker Selection	ORR, %	Median PFS, Months	PFS, HR (95% CI)	OS, HR (95% CI)	Grade 3 or Higher AEs, %	References
ADCs										
NCT03784599 (TRAEMOS)	2	Trastuzumab emtansine (HER2 ADC) + osimertinib		HER2 overexpression	4 (*n* = 27)	2.8			41	[120]
NCT04619004 (HERTHENA-Lung01)	2	Patritumab deruxtecan (HER3 ADC)		None	30 (*n* = 225)	5.5			65	[116]
NCT05338970 (HERTHENA-Lung02)	3	Patritumab deruxtecan (HER3 ADC)	Osimertinib	None						[117]
NCT04676477	1	Patritumab deruxtecan (HER3 ADC) + osimertinib		None						[121]
NCT03539536	2	Telisotuzumab vedotin (MET ADC)		MET overexpression	12 (*n* = 43)					[122]
NCT02099058	1	Telisotuzumab vedotin (MET ADC) + erlotinib or osimertinib		MET overexpression	27 (*n* = 15) ^d^				64	[123]
NCT04484142 (TROPION-Lung05)	2	Datopotamab deruxtecan (TROP2 ADC)		None	44 (*n* = 78)				48	[124]
Chemoimmunotherapy										
NCT03515837 (KEYNOTE789)	3	Pembrolizumab + platinum + pemetrexed	Platinum + pemetrexed	None	29 (*n* = 245) vs. 27 (*n* = 247)	5.6 vs. 5.5	0.80 (0.65–0.97)	0.84 (0.69–1.02)	44 vs. 39	[101]
NCT02864251 (CheckMate722)	3	Nivolumab + platinum + pemetrexed	Platinum + pemetrexed	None	31 (*n* = 144) vs. 27 (*n* = 150)	5.6 vs. 5.4	0.75 (0.56–1.00)	0.82 (0.61–1.10)	45 vs. 29	[100]
NCT03991403 (ATTLAS) ^e^	3	Atezolizumab + bevacizumab + platinum + pemetrexed	Platinum + pemetrexed	None	70 (*n* = 151) vs. 42 (*n* = 74)	8.5 vs. 5.6	0.62 (0.45–0.86)	1.01 (0.69–1.46)	35 vs. 15	[104]
NCT03802240 (ORIENT-31)	3	Sintilimab + platinum + pemetrexed	Chemotherapy	None	55 (*n* = 158) vs. 47 (*n* = 160)	5.5 vs. 4.3	0.72 (0.55–0.94)	0.98 (0.72–1.34)	56 vs. 49	[102]
		Sintilimab + IBI305 (bevacizumab biosimilar) + platinum + pemetrexed	Chemotherapy	None	65 (*n* = 148) vs. 27 (*n* = 160)	7.2 vs. 4.3	0.51 (0.39–0.67)	0.97 (0.71–1.32)	41 vs. 49	[102]

^d^, reported in the cohort treated with telisotuzumab vedotin (MET ADC) + erlotinib. ^e^, including EGFR- or ALK-mutated NSCLC. EGFR, epidermal growth factor receptor; mAb, monoclonal antibody; NSCLC, non-small cell lung cancer; TKIs, tyrosine kinase inhibitors; ORR, overall response rate; PFS, progression-free survival; HR, hazard ratio; Cl, confidence interval; OS, overall survival; AEs, adverse events; ADC, antibody–drug conjugate; ALK, anaplastic lymphoma kinase; RET, rearranged during transfection; MET, mesenchymal–epithelial transition; CNS, central nervous system; HER2, human epidermal growth factor receptor 2; FISH, fluorescence in situ hybridization; NGS, next-generation sequencing; GCN, gene copy number.

## Data Availability

The data presented in this study are available upon request from the corresponding author.
